# Influence of the Nature and Structure of Polyelectrolyte Cryogels on the Polymerization of (3,4-Ethylenedioxythiophene) and Spectroscopic Characterization of the Composites

**DOI:** 10.3390/molecules27217576

**Published:** 2022-11-04

**Authors:** Svetlana Laishevkina, Natalia Shevchenko, Olga Iakobson, Anatoly Dobrodumov, Vladimir Chelibanov, Elena Tomšík

**Affiliations:** 1 Institute of Macromolecular Compounds, Russian Academy of Sciences, Bolshoy pr., 31, St. Petersburg 199004, Russia; 2 Department of Environmental Instrumentation and Monitoring, State University of Information Technologies, Mechanics and Optics, Kronverksky Pr. 49, bldg. A, St. Petersburg 197101, Russia; 3 Institute of Macromolecular Chemistry AS CR, Heyrovsky Sq. 2, 162 00 Prague, Czech Republic

**Keywords:** polyelectrolyte gels, composite PEDOT@polyelectrolyte, electrochemical impedance spectroscopy

## Abstract

Conductive hydrogels are polymeric materials that are promising for bioelectronic applications. In the present study, a complex based on sulfonic cryogels and poly(3,4-ethylenedioxythiophene) (PEDOT) was investigated as an example of a conductive hydrogel. Preparation of polyacrylate cryogels of various morphologies was carried out by cryotropic gelation of 3-sulfopropyl methacrylate and sulfobetaine methacrylate in the presence of functional comonomers (2-hydroxyethyl methacrylate and vinyl acetate). Polymerization of 3,4-ethylenedioxythiophene in the presence of several of the above cryogels occurred throughout the entire volume of each polyelectrolyte cryogel because of its porous structure. Structural features of cryogel@PEDOT complexes in relation to their electrochemical properties were investigated. It was shown that poly(3,4-ethylenedioxythiophene) of a linear conformation was formed in the presence of a cryogel based on sulfobetaine methacrylate, while minimum values of charge-transfer resistance were observed in those complexes, and electrochemical properties of the complexes did not depend on diffusion processes.

## 1. Introduction

Conductive polymer hydrogels are a unique class of materials that combine the beneficial properties of hydrogels and organic conductors; they are used in many applications such as bioelectronics [1,2,3]. Among a large number of conductive polymers, poly(3,4-ethylenedioxythiophene) (PEDOT) is of particular interest [1] due to its chemical stability and optical transparency in the oxidized state, biocompatibility [4], and good electrical activity in phosphate buffers [5]. The main disadvantages of PEDOT are its insolubility in solvents and its poor mechanical properties. An improvement of PEDOT mechanical properties can be achieved by preparing it as part of composites with templates of various structures: soluble and film-forming polymers [6,7,8], latex particles [9,10,11], etc. The most promising templates are sulfonated polymers, which form stable complexes with PEDOT [12].

Preparation of materials for flexible bioelectronics suggests a polymer template, mostly often a hydrogel, to be synthesized as a first stage [13,14,15]. Next, an electrically conductive polymer is formed in the structure of this template [16]. A number of requirements are imposed on polymer gel templates; in particular, this gel must have a developed system of pores and be elastic [17,18,19]. However, synthesis of rigid-chain conjugated electrically conductive polymers in such soft gels is a difficult task. One of the difficulties lies in the fact that oxidizers (for example, iron salts) are used for synthesis of biocompatible electrically conductive gels. Removal of oxidation products from resulting electrically conductive gels is a time-consuming and sometimes difficult task. As a result, obtained electrically conductive gels cannot be further investigated as bioelectronic materials.

The mentioned electrically conductive gel was first obtained by Gilmore et al. [20] during pyrrole electropolymerization in the structure of polyacrylamide hydrogel. Electrically conductive gels based on PEDOT complexes have been intensively studied over the last 10 years [21,22,23,24]. Common templates used as hydrogels include natural or synthetic gels based on agarose [25], alginate [26], fibrin [27], polyurethane [28], and polyacrylic acids [29].

It was shown that confined polymerization of 3,4-ethylenedioxythiophene (EDOT) inside nanopores of mesoporous silica favors formation of a more linear configuration of PEDOT polymer chains, which is typical of PEDOT-based complexes after secondary doping with organic solvents [30]. Formation of a linear PEDOT structure allowed PEDOT chains to be oriented relative to each other with small torsion angles. As a result, crystalline order of PEDOT chains increased, leading to improvement of electrochemical properties of films based on PEDOT/template complexes.

Currently, investigations are being carried out to find methods of synthesis of suitable gels with desired properties (developed pore structure, limited swelling, good mechanical properties) and methods for polymerization of thiophene derivatives in them, which would make it possible to avoid time-consuming purification procedures.

One possible method is to carry out EDOT polymerization in the presence of Brønsted acids [31]. In [32], the synthesis of a number of conducting polymers was first carried out in the presence of formic acid without explicit addition of an oxidizer. At the same time, it should be noted that the exact mechanism of EDOT polymerization in the presence of formic acid without explicit use of an oxidizer (the method used as a method for the synthesis of PEDOT herein) has not yet been established. In addition, analysis of the literature shows that there are no data describing in detail EDOT polymerization under the action of formic acid in the presence of sulfocontaining gels.

In our work, cryogels based on 3-sulfopropyl methacrylate and sulfobetaine methacrylate were used as a structured polymer template with a developed pore system. We have shown for the first time that structure and nature of obtained cryogels have a significant effect on EDOT polymerization under action of formic acid. During this study, the effect of porosity of the cryogel on confined polymerization of EDOT and structure of the resulting cryogel@PEDOT complex were established. Structure of the cryogel@PEDOT composites was confirmed by Raman-, FTIR-, and ^13^C-NMR Solid State spectroscopy and studied by scanning electron microscopy (SEM). Perspectives of the complexes obtained for the bioelectronic applications were studied by electrochemical impedance spectroscopy.

## 2. Results and Discussion

### 2.1. Preparation of Polyelectrolyte Cryogels Based on Sulfonic Monomers

Use of polyelectrolyte templates with a developed specific surface for PEDOT synthesis was assumed to lead to a more uniform distribution of conductive polymer in the template and promote the creation of more prolonged charge transport channels in the polymer composite. From this point of view, preparation of polyelectrolyte cryogels based on sulfonic monomers was of specific interest.

Polyelectrolyte gels were obtained in the process of cryotropic gelation. Potassium 3-sulfopropyl methacrylate (SPM) and potassium sulfobetaine methacrylate (SBMA) were used as sulfocontaining monomers. Polymerization of sulfocontaining monomers was carried out in the presence of a cross-linking agent: N,N′-methylenebis(acrylamide) (MBA) at a temperature of −18 °C. The process of gel formation at temperatures below the solvent crystallization point is usually called “cryotropic gelation” [33]. In that case, the reaction proceeds in a concentrated nonfreezing microphase around ice crystals, which act as a porogen and ensure the formation of a gel with a highly porous structure after thawing. An advantage of cryotropic gelation is ability to control the diameter and structure of pores that modify freezing conditions [34].

Gel samples obtained in this study are described in Table 1.

Gel intended for use as a structure-forming template for EDOT polymerization should have a minimum degree of swelling in various solvents. This contributes to dense packing of PEDOT chains in those structures and formation of more extended conjugated systems. Good mechanical properties, as well as a developed pore structure, make it possible to improve performance properties and expand the scope of those gels. Previously, we described in detail the conditions for formation and properties of cryogels based on SPM and SBMA [34]. Optimal conditions for formation of cryogels, which make it possible to obtain gels with desired characteristics, are shown in Table 2. It was shown that a necessary condition for formation of cryogels with desired properties was introduction of 50 mol.% of comonomers (2-hydroxyethyl methacrylate (HEMA) or vinyl acetate (VA)) and 1 mol.% of crosslinking agent (N,N′-methylene-bis-acrylamide, MBA).

**Figure 1:**
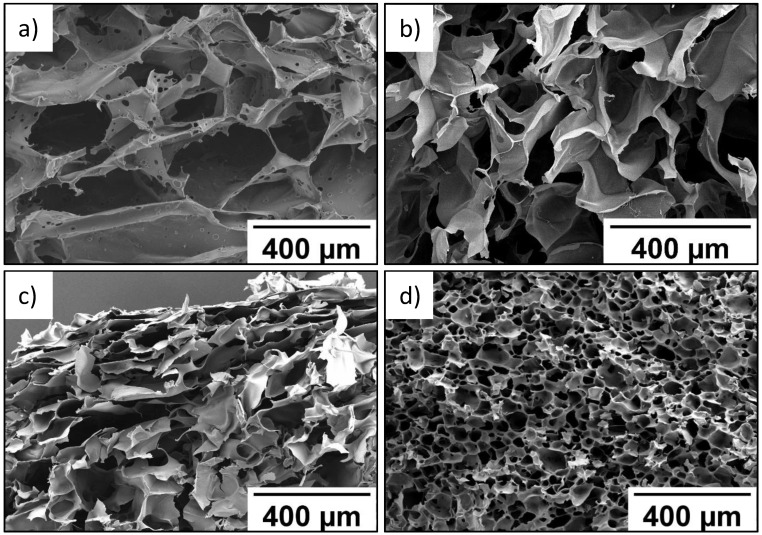
SEM images of P(SPM-VA) (**a**), P(SPM-HEMA) (**b**), P(SBMA-HEMA) (**c**), and P(SBMA-VA) (**d**) gels.

As seen in SEM data, wall thickness of the P(SPM-VA) cryogel ranged from 10 to 40 µm, while pore size varied from 210 to 360 µm (Figure 1a). At the same time, introduction of HEMA led to formation of the P(SPM-HEMA) cryogel with a pore size of 110–170 µm (Figure 1b).

Structure of the walls of cryogels based on the SBMA copolymer was more uniform in thickness, with wall thickness being 10–15 µm, while pores of the smallest size (30–40 µm) were typical for the P(SBMA-VA) cryogel (Figure 1c). Uniform distribution of pores over the entire volume was also observed for the P(SBMA-VA) cryogel, which led to an increase in specific surface area by more than four times (Table 2). Moreover, mesopores of minimum sizes (4–5 nm) were turned to form in the P(SBMA-VA) cryogel [34], while mesopore size ranged from 8–15 nm for cryogels based on SPM copolymers. As a result of significant decrease of pore size, degree of swelling of the P(SBMA-VA) cryogel in formic acid was minimal and did not exceed 43 g/g (Table 2). Uniform distribution of relatively small pores over the entire volume of the P(SBMA-VA) cryogel ensured its good mechanical properties [34]. Therefore, gels were characterized by presence of two types of pores: pores in place of ice crystals, with a diameter of 30–350 µm, and mesopores in the polymer walls of the gels, with a diameter of 4–15 nm.

**Figure 2:**
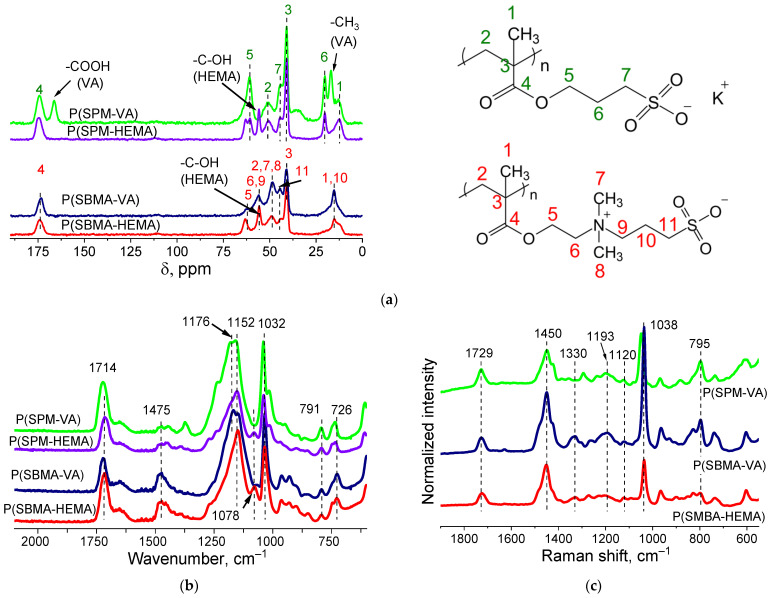
CP/MAS ^13^C NMR (**a**), ATR-FTIR (**b**), and Raman Spectra (**c**) of P(SPM-VA), P(SPM-HEMA), P(SBMA-VA), and P(SBMA-HEMA) gels.

Structure of the gels was proven by ATR-FTIR- and Raman- and CP/MAS ^13^C-NMR–spectroscopy studies (Figure 2). CP/MAS ^13^C-NMR spectra of dried cryogels P(SPM-VA) and P(SPM-HEMA) (Figure 2a) showed peak characteristics of SPM: chemical shifts at 175, 61–63, 43, and 20 ppm, related to the carbon atom of the carboxyl group, the carbon associated with oxygen, a sulfogroup, and an intermediate carbon (attached to two other carbon atoms), respectively. This is consistent with data in the literature. For P(SBMA-VA) and P(SBMA-HEMA) samples, chemical shifts characteristic of SBMA were observed: 175, 63, 56, and 41 ppm, related to the carbon atom of the carboxyl group and the sulfogroup as well as to the carbon associated with oxygen and the middle carbon (attached to two other carbons), respectively. Moreover, the NMR study confirmed presence of comonomers. Chemical shifts at 166 and 18 ppm, related to the carbon atom of the carboxyl and methyl groups of VA, were observed, and presence of HEMA in the structure of the polymer chains was confirmed by the peak at 63 ppm.

ATR-FTIR spectra (Figure 2b) showed the following bands: 1714 cm^−1^ (stretching vibration −C=O), 1475 cm^−1^ (δ_s_ CH_2_), 1165 cm^−1^ (ν_as_ -C-C(=O)-O), 1155 cm^−1^ (ν_as_ O-C-C), 1032 cm^−1^ (ν_s_ S=O), 791 cm^−1^ (ν -C-S-O), and 726 cm^−1^ (ρ CH_2_) (Figure 2b). In addition, there was a band at 1078 cm^−1^ that corresponded to –C–OH of the HEMA comonomer in the case of the P(SBMA-HEMA) gel, and a band at 1373 cm^−1^ related to symmetric bending CH_3_ vibrations of vinyl acetate in the case of P(SPM-VA). This confirmed presence of comonomers in the structure of the gels.

Raman spectra (Figure 2c) showed characteristic bands at 1038, 1120, and 1193 cm^−1^, belonging to stretching vibrations of the sulfonate ion −SO_3_^−^ and the undissociated sulfogroup −SO_3_H, as well as symmetrical stretching vibrations of SO_2_. In addition, characteristic modes were observed at 606 cm^−1^ (ν C-C(=O)-O + ν_s_ C-C-O), 795 cm^−1^ (ν C-S), 1294 cm^−1^ (ν C-C(=O)-O), 1423 cm^−1^ (δ_as_ CH_3_), 1449 cm^−1^ (δ CH_2_), and 1727 cm^−1^ (ν C=O).

**Figure 3:**
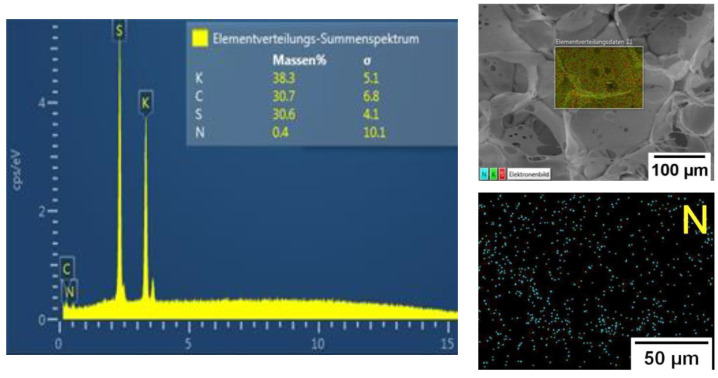
EDX analysis data for the P(SPM-VA) cryogel.

NMR, FTIR, and Raman spectroscopy studies failed to show presence of MBA in the gel structure, which may be due to its low content in the reaction system. However, high content of the sol-gel fraction (Table 2, more than 75%) measured in hot water indicated presence of chemical crosslinking in the gels. In addition, EDX analysis of P(SPM-VA) (Figure 3) showed a small content of N (~0.4 wt%), which was evenly distributed throughout the gel structure, confirming presence of MBA units in the gel. It should be noted that in the case of SBMA gels, this method was not conclusive, since nitrogen was a part of the monomer unit of both SBMA and MBA (see Supplementary Materials).

Concentration of sulfogroups in obtained polyelectrolyte cryogels was studied. The value of ion-exchange capacity for the P(SPM-VA) cryogel was equal to 2.3∙10^−4^ mol/g, which was a bit lower than the maximum value of the ion-exchange capacity obtained for the cross-linked PSPM cryogel containing no functional comonomer units (3∙10^−4^ mol/g) [34]. It was not possible to estimate concentration of sulfogroups in zwitterionic polymers based on SBMA by measuring their ion-exchange capacity, since both cations and anions contained in the structure of each monomer unit participated in ion exchange.

### 2.2. Investigation of EDOT Polymerization in the Presence of Polyelectrolyte Cryogels

In this work, we studied a process of cryogel@PEDOT complex formation during EDOT polymerization in the presence of formic acid (without the use of oxidizing agents), using a cryogel template. EDOT polymerization was carried out in the presence of HCOOH (98%); after the monomer was dissolved in ethanol, the reaction proceeded for 10 days at room temperature [35]. Generally, the mechanism of EDOT polymerization is well-established. First, the EDOT monomer is oxidized to cationic radicals, followed by free radical dimerization. The achieved dimer consequently experiences a deprotonation process, resulting in an active neutral dimer, which facilitates the dimer to react in the following oxidation process for chain growth [36]. At the same time, it should be noted that the exact mechanism of EDOT polymerization in the presence of formic acid without explicit use of an oxidizer (the method used for PEDOT synthesis in our work) has not yet been established. The authors of [32] suggested a possible route for self-polymerization of EDOT in the presence of formic acid. However, in our case, we could not exclude the participation of air oxygen dissolved in the reaction system, including that captured by cryogel pores, in EDOT oxidation. Therefore, the general mechanism of EDOT polymerization in our work was assumed not to fundamentally differ from that described in the literature. At the same time, as in the work of [32] in the case of polymerization of EDOT in a cryogel template in the presence of formic acid, the latter is likely to have electrocatalytic properties that activate the monomer and decrease its oxidation potentials due to a shift of C-α electron density in the thiophene ring of EDOT.

Among obtained cryogels, P(SPM-HEMA) was not suitable for synthesis of PEDOT, since the gel was characterized by a high swelling ratio in water (see Table 2). EDOT polymerization in the presence of P(SPM-VA) cryogel resulted in gel destruction during the process. As shown in [34], stabilization of the P(SPM-VA) cryogel structure was mainly ensured by ion–ion interaction. During EDOT polymerization, the cryogel was destroyed as a result of the breaking of those stabilizing ionic bonds due to participation of ionized cryogel groups in interaction with the growing PEDOT chain. At the same time, the P(SBMA-HEMA)@PEDOT and P(SBMA-VA)@PEDOT complexes were stable and were destroyed neither during their washing from the reaction products nor during subsequent studies by electrochemical impedance spectroscopy.

EDOT polymerization, both in the presence of polyelectrolyte cryogels and without them, was accompanied by a color change from colorless to dark blue, which is typical for PEDOT formation. The color change was associated with the formation of a π-conjugated system. It is known that absorption spectra in the visible and near IR regions of PEDOT are characterized by three absorption bands with absorption maxima at 600, 850, and >1100 nm [37]. Absorption at λmax = 600 nm was due to π→π* electronic transitions in the reduced state of PEDOT. Two other absorption bands at λmax = 850 and >1100 nm corresponded to the oxidized state of PEDOT [37].

**Figure 4:**
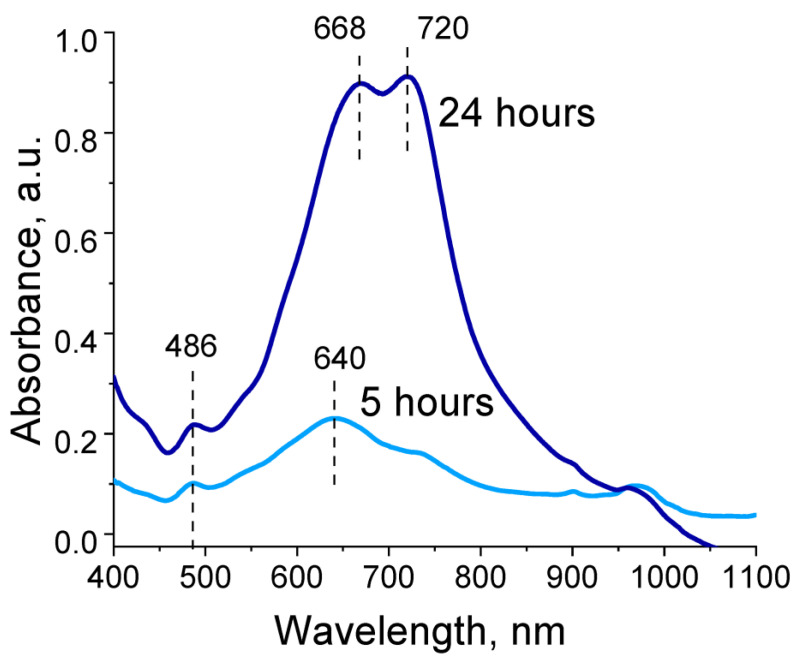
Optical absorption spectra of reaction dispersions during EDOT polymerization in the presence of P(SBMA-VA).

Absorption spectra recorded during EDOT polymerization in the presence of polyelectrolyte cryogels exhibited two absorption bands: a peak at 480 and a peak with two maxima in the range of 670–720 nm. Intensity of peaks continued to increase for 48 h of EDOT polymerization (Figure 4). These absorption bands corresponded to the reduced state and one of the oxidized states of PEDOT (polaron). In our case, hypsochromic shift of both absorption bands relative to those characteristic of PEDOT could be observed, associated with a decrease in the length of the π-conjugated system [38]. Thus, during EDOT polymerization in the presence of the obtained polyelectrolyte templates, growth of long PEDOT chains was hindered and structures with high charge localization were formed. Presence of two maxima for the absorption band in the region of 670–720 nm is likely explained by presence of two types of pores in the cryogel structure, in which independent polymerization of EDOT seemed to occur with differing formation of PEDOT in the length of the polymer chain.

**Figure 5:**
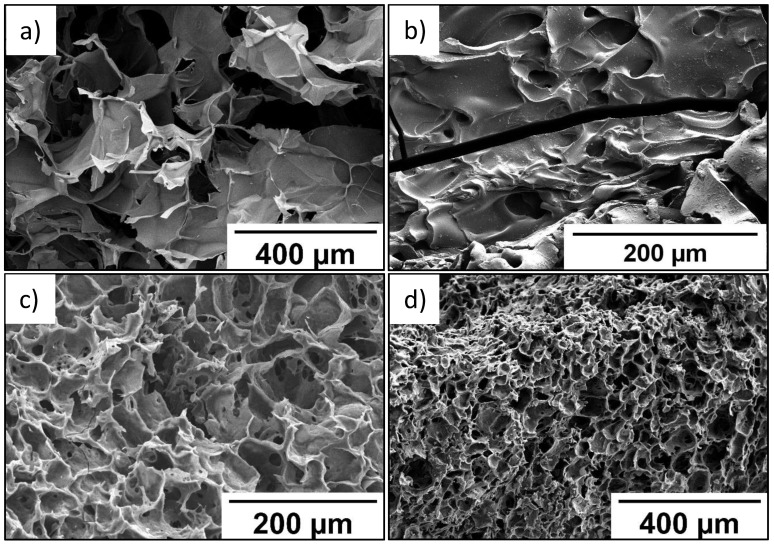
SEM images of films based on P(SBMA-HEMA)@PEDOT (**a**,**b**) and P(SBMA-VA)@PEDOT (**c**,**d**).

SEM data (Figure 5) indicate that the P(SBMA-HEMA)@PEDOT and P(SBMA-VA)@PEDOT complexes retained uniform pore distribution over the entire volume. It should be noted that the images in Figure 5a,c,d were obtained for freeze-dried P(SBMA-HEMA)@PEDOT and P(SBMA-VA)@PEDOT complexes. Therefore, in aqueous systems, complexes based on P(SBMA-HEMA)@PEDOT and P(SBMA-VA)@PEDOT are permeable to solvent molecules. Thickness of the walls of the obtained complexes decreased on average by ~50% compared to initial cryogels without PEDOT. Furthermore, degree of swelling in formic acid significantly decreased to 8 and 1.5 g/g for the P(SBMA-HEMA)@PEDOT and P(SBMA-VA)@PEDOT complexes, respectively. Obviously, this indicates a decrease in the free internal volume of complexes based on cryogels due to ion–ion interactions between PEDOT chains and the polyelectrolyte template; thus, PEDOT polymerization occurs both on the surface and in the volume of polyelectrolyte gels.

**Figure 6:**
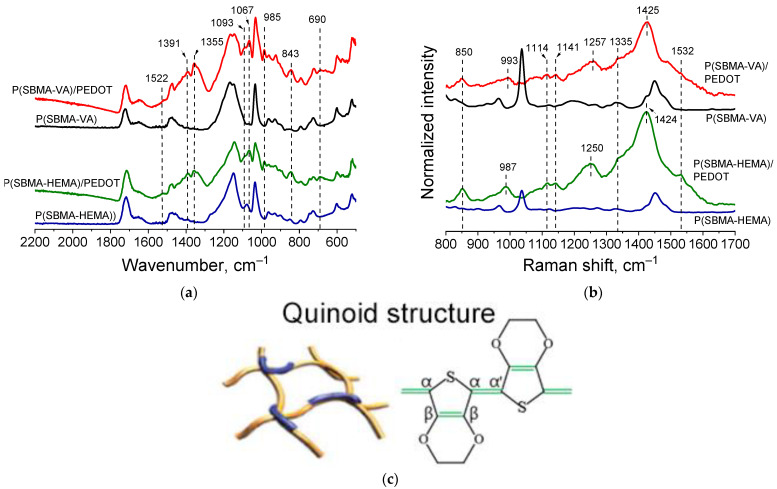
FTIR (**a**) and Raman (**b**) spectra for cryogel complexes with PEDOT; proposed structure of PEDOT in complexes (**c**).

Presence of PEDOT in the structure of the cryogel@PEDOT complex was confirmed by ATR-FTIR and Raman spectroscopy studies. FTIR spectra (Figure 6a) showed characteristic bands at 1093, 1067 cm^−1^, corresponding to C-O-C stretching vibrations of ethylenedioxygroups. Bands at 985, 843, and 690 cm^−1^ corresponded to the stretching vibrations of the C-S-C bond in the thiophene ring. Bands at 1522 and 1355 cm^−1^ referred to asymmetric stretching vibrations of the C=C and C–C bonds in the thiophene ring, respectively [30]. In addition, it could be observed that absorption in the region above 2000 cm^−1^, the so-called “conductivity plateau” [38], was present; it was absent in the spectrum of the initial cryogels. Obtained spectra proved formation of PEDOT in the structure of cryogels.

Figure 6b (red and green spectra) shows Raman spectra of the obtained complexes. Characteristic bands—in particular the oxyethylene ring deflection band at 987 cm^−1^, the C-O-C deflection band at 1114 cm^−1^ and at 1141cm^−1^, the inter-ring Cα–Cα’ stretching band at 1257 cm^−1^, and the Cβ–Cβ stretching band at 1335cm^−1^—could be clearly detected. The characteristic band at 1425 cm^−1^ due to symmetric Cα = Cα (−O) stretching was indicative of a high level of conjugation in the structure of PEDOT [39]. It should be noted that presence of a characteristic band at 1425 cm^–1^ indicated formation of a polaron in the PEDOT structure (sometimes referred to in the literature as “quinoid structure of PEDOT”). At the same time, another characteristic PEDOT band in the region of 1550–1570 cm^−1^ corresponding to bipolaronic PEDOT structure was not observed for cryogel@PEDOT complexes. This correlated with results obtained during the study of optical absorption spectra in the visible and near-IR regions (Figure 4).

As can be seen from the spectrophotometric data presented above, small PEDOT chains were formed as a result of PEDOT synthesis in the cryogel template. Taking into account the results of Raman spectroscopy investigation of obtained cryogel@PEDOT complexes together with data in the literature regarding confined EDOT polymerization [30] and polar Bronsted acid-assisted EDOT polymerization [35], we could assume a linear (extended) conformation of PEDOT in the present study.

It should be noted that having a length smaller than the polymer persistence length was an important requirement for polymer chains to be effective for carrier transport. If this requirement is satisfied, chains will not bend significantly, therefore retaining electronic conjugations [30]. Linear chain conformation assumed for PEDOT here suggested the possibility of π–π stacking between PEDOT chains, leading to efficient charge-transport properties according to the interchain charge transport model. Undoubtedly, this assumption requires further studies of electrical properties of resulting cryogel@PEDOT complexes. Therefore, presence of a gel based on SBMA copolymers as a polyelectrolyte component, as well as presence of pores in the polymer walls of the gel, made it possible to form PEDOT with a linear conformation. This led to better interaction of PEDOT chains with each other and delocalization of conjugated π-electrons, which also corresponded to the results of FTIR spectroscopy, in which P(SBMA-HEMA)@PEDOT and P(SBMA-VA)@PEDOT complexes formed had a conductivity plateau in the region of 1800–2000 cm^−1^.

**Figure 7:**
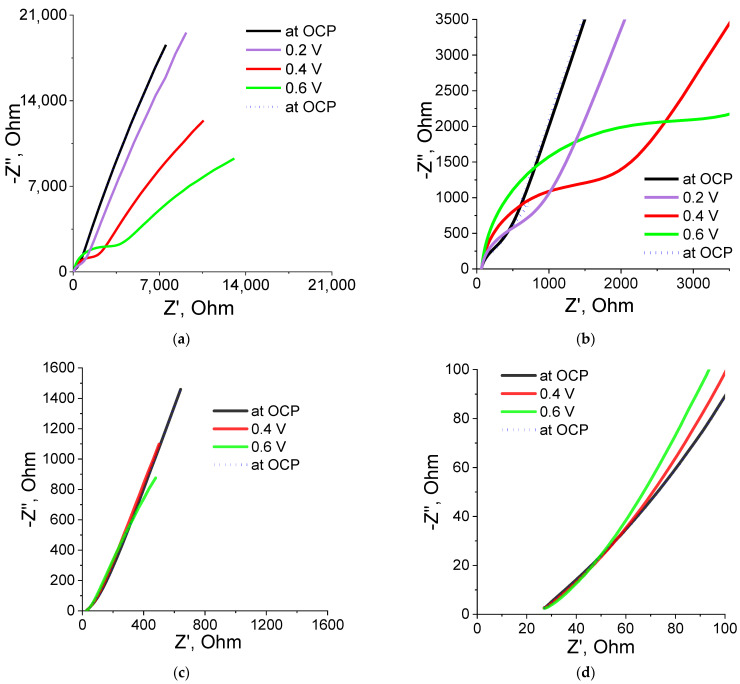
EIS spectra and their enlarged part in the high-frequency region for the P(SBMA-HEMA)@PEDOT (**a**,**b**) and P(SBMA-VA)@PEDOT (**c**,**d**) complexes.

P(SBMA-HEMA)@PEDOT and P(SBMA-VA)@PEDOT complexes obtained were studied by electrochemical impedance spectroscopy (EIS). Spectra were taken in the frequency range of 0.1 Hz–10 kHz in the region of their electrical activity (0.2–0.6 V), as well as at the open circuit potential (OCP). The spectrum in the Nyquist coordinates (dependence of the imaginary on the real part of the impedance) had three types of characteristic sections: semicircles in the higher frequency region, direct diffusion impedance, and vertical capacitive response (Figure 7). Calculation of resistance values (Rct) was carried out in an analysis of experimental data fitting using the FRA32M module and Nova 2.1 software that used the Randles scheme. The confidence interval (χ^2^) for all measurements was <0.3.

Figure 7a,b show the spectrum and an enlarged part of the spectrum of the P(SBMA-HEMA)@PEDOT complex. The enlarged part clearly shows presence of part of the semicircle at high frequencies. This indicates that, at those frequencies, the predominant contribution to the impedance spectrum was made by resistance to charge transfer at the electrode–electrolyte interface. In this case, measurement of EIS at different potentials led to an increase in charge-transfer resistance. In addition, during measuring of EIS at different potentials, an increase in slope of the direct spectrum was observed. This indicated an increase in the contribution of the diffusion component of the impedance (Warburg impedance). As a result, one could state that this type of complex strongly depended on diffusion processes, which may be due to the fact that both the initial P(SBMA-HEMA) template and the P(SBMA-HEMA)@PEDOT complex had a high degree of swelling (50 g/g).

The enlarged part of the spectrum of the P(SBMA-VA)/PEDOT complex (Figure 7d) demonstrates the absence of a semicircle, which means that for this complex, there was practically no resistance to charge transfer (Rct at 0.6 V was 9.28 Ω, χ^2^ = 0.19 (data on the simulation circuit of Rct in EIS analysis are given in Supplementary Figure S6)). In addition, for this complex, neither charge-transfer resistance nor Warburg diffusion impedance was practically independent of applied potential in the range of 0.2–0.6 V; on the EIS spectrum (Figure 7c,d), all spectra practically overlapped each other. It is also worth noting that both complexes returned to their original properties, which indicated good reproducibility of their properties as well as absence of sample deformations during the measurement process. These conclusions were confirmed by the results obtained by cyclic voltammetry. Stable behavior of the films was observed both for the P(SBMA-HEMA)@PEDOT and P(SBMA-VA)@PEDOT complexes during repeated cycling in the potential range from –0.1 to 0.8 V vs. Ag/AgCl at a sweep rate from 10 to 150 mV/s (see Supplementary Materials).

## 3. Materials and Methods

### 3.1. Chemicals

Sulfobetaine methacrylate (SBMA) (Sigma-Aldrich, Saint Louis, MO, USA), 3-sulfopropyl methacrylate potassium salt (SPM) (Sigma-Aldrich, Saint Louis,, MO, USA), 2-hydroxyethyl methacrylate (HEMA) (Sigma-Aldrich, Saint Louis,, MO, USA), N,N′-methylenebis(acrylamide) (MBA) (Sigma-Aldrich, Saint Louis, USA), 3,4-ethylenedioxythiophene (EDOT) (Sigma-Aldrich, Saint Louis,, MO, USA), formic acid (Sigma-Aldrich, Moscow, Russian federation), and H_2_SO_4_ (Vecton, St. Petersburg, Russian federation) were used without preliminary purification. Vinyl acetate (VA) (Sigma-Aldrich, Moscow, Russian federation), potassium persulfate (K_2_S_2_O_8_) (Vecton, St. Petersburg, Russian federation), N,N,N′,N′-tetramethyl ethylenediamine (TEMED) (Vecton, St. Petersburg, Russian federation), sodium chloride (Vecton, St. Petersburg, Russian federation), HCl (Vecton, St. Petersburg, Russian federation), and ethanol (Vecton, St. Petersburg, Russian federation) were purchased from Vekton LCC (Russia). K_2_S_2_O_8_ was purified through recrystallization from ethanol. VA was purified through vacuum distillation according to standard methods.

Bidistilled water was used to prepare solutions and carry out polymerization. Water was twice-distilled from an all-glass apparatus. Water-specific conductivity (0.2 S/cm) and surface tension (72 mN/m) proved that it was free of surface-active impurities.

To measure ion-exchange capacity and degree of swelling of the polyelectrolyte matrix, aqueous solutions of NaCl (Vecton, St. Petersburg, Russian federation), as well as hydrochloric acid (HCl) (Vecton, St. Petersburg, Russian federation) and formic acid (HCOOH, Sigma-Aldrich, Moscow, Russian federation), were used as received. Solutions of NaOH (Sigma-Aldrich, Moscow, Russian federation) were prepared by dissolving alkali crystals in bidistilled water after they were washed from sodium bicarbonate. These solutions were used over 3 days. Their exact concentrations were determined by conductometric titration with a standard HCl solution. HCl solutions were prepared from standard titers.

### 3.2. Preparation of Cryogels

Initial reaction solutions contained 0.3 mmol of sulfocontaining monomers (SPM or SBMA), 0.3 mmol of comonomer (HEMA or VA), 1 mol.% to Mn of MBA, 1 mL of water, and the initiator system (K_2_S_2_O_8_ 1 mol.% to monomer Mn and TEMED 2 mol.% or 10 mol.% to Mn.) Synthesis of cryogels was carried out according to the following procedure: Initial reaction solutions were prepared within 2 min at room temperature in a plastic syringe and kept in a freezer at −18 °C for 1 day. Next, syringes were taken out of the freezer and brought to room temperature; resulting cryogels were washed 3 times with the excess amount of water in a 600 mL beaker to remove unbounded reaction products. Finally, cryogels were freeze-dried before further use.

### 3.3. Polymerization of EDOT in the Presence of Cryogels

Synthesis of 3,4-ethylenedioxythiophene (EDOT) was carried out according to the following procedure:

In a bottle, 56 μL (0.53 mmol) of EDOT and 2 mL of ethanol were mixed, after which 4.6 mL of HCOOH (>95%) was added (HCOOH/EDOT ratio = 75, *w*/*w*). Next, 0.1 g of the polymer template was placed into the resulting solution. The reaction was carried out at room temperature for 10 days. After that, polyelectrolyte complexes were washed with ethanol.

### 3.4. Characterizations of Cryogels and Cryogel@PEDOT Structure by SEM and EDX

SEM images of cryogels and cryogel@PEDOT were obtained using field emission scanning electron microscopy (Hitachi S4800 FESEM, Tokyo, Japan). Pore structure of obtained hydrogels was studied in the swollen state. For this purpose, cryogel samples were exposed to water until equilibrium was established, then frozen rapidly and lyophilized. Freeze-dried pieces of cryogel were placed on a silicon wafer, then given sputtering with 10 nm of AuPd. Afterwards, the sample was inserted into an observation electron microscope chamber.

Elemental composition of gels was examined by an energy-dispersive X-ray (EDX) attached to the SEM (Zeiss—SUPRA 55) with a high-sensitivity X-ray detector.

### 3.5. Ion Exchange Capacity

Ion exchange capacity (IEC) of the synthesized polyelectrolyte matrix was measured by acid-base titration. Typically, 0.1 g of sample was treated with 10 mL of 0.5 M HCl and stirred for 1 day. The sample was then dialyzed to remove any excess of acid or salt. After freeze-drying, the sample was treated with 20 mL of 20% NaCl solution for 6h to exchange all H^+^ ions with Na^+^. HCl formed as a result when this ion exchange was titrated with 0.01 M NaOH, using phenolphthalein as the indicator. Ion exchange capacity was calculated using the following equation:

$$Q=C_{T}\frac{V_{T}}{m_{\mathrm{gel}}}$$

where C_T;_—titrant concentration, V_T;_—titrant volume, and m_gel_—the weight of the polyelectrolyte template.

### 3.6. Determination of Gel Fraction

Gel fraction of the polyelectrolyte template was determined by continuous Soxhlet extraction [39]. Extraction was carried out in DI water at 100 °C within 4 h. After extraction, the remaining polymer was freeze-dried.

### 3.7. Determination of Specific Surface Area ([S])

Specific surface area and pore size distribution of synthesized cryogels were assessed by a nitrogen gas sorption analyzer (NOVA 1200, Quantachrome, Houston, USA) and determined by a Multipoint BET (Brunauer–Emmett–Teller) method. The DFT (density functional theory) method was applied to calculate pore size distribution from analysis of desorption branches of the isotherms. Preliminarily, water was removed from cryogels by evaporation at a temperature of 40 °C under reduced pressure using a Laborota4011 rotary evaporator. The sample was degassed before measurement of nitrogen flow under reduced pressure.

### 3.8. ATR-FTIR, CP/MAS ^13^C NMR, Raman, and Vis-NIR Spectroscopy

Since both the initial cryogels and their complexes with PEDOT were insoluble, spectral studies were carried out for freeze-dried powders.

FTIR spectra of the samples were recorded in the range of 500–4000 cm^−1^ using an attenuated total reflectance (ATR) attached FTIR spectrometer IR-Affinity-1S (Shimadzu, Kyoto, Japan). All spectra represented an average of 32 scans taken.

Raman spectra of samples were recorded on OPTEC-785-H and OPTEC-785-SL (Optec, St. Petersburg, Russia) analytical systems with an excitation wavelength of 785 nm. Measurements were carried out in the range of 3000–300 cm^−1^.

Structure of the polyelectrolyte template was investigated by solid state CP/MAS ^13^C NMR spectroscopy (Cross-Polarization Magic-Angle-Spinning NMR). NMR spectra were recorded on an AVANCE II-500 WB NMR spectrometer (Bruker, Bochum, Germany) operating at a resonance frequency of 125.8 MHz for ^13^C NMR. Polymer samples were packed into zirconium rotors with a diameter of 4 mm; spectra were registered at a temperature of 20 °C and a rotation frequency of 10 or 13 kHz.

Vis-NIR absorption spectra were recorded using a LOMO SF-56 (LOMO Microsystems, St. Petersburg, Russia). Spectra were registered in the range of 400 to 1100 nm.

### 3.9. Electrochemical Impedance Spectroscopy (EIS)

Electrochemical impedance spectroscopy measurements were carried out in a three-electrode cell. FTO glasses were used as working electrodes, Pt plate (1.2 cm^2^) was used as an auxiliary electrode, and Ag/AgCl (3 M KCl) was used as a reference electrode. For data recording, an AUTOLAB PGSTAT302N potentiostat with an FRA32M module and Nova 2.1 software was used. Measurements were carried out at a temperature of 25 °C in the frequency range from 10 kHz to 1 Hz at different potentials. A Kroning–Kramers test was applied to verify obtained EIS data. Before conduction of electrochemical studies, the working electrode was immersed in a supporting electrolyte for several hours.

Resulting cryogel@PEDOT complexes were applied to the surface of the working electrode. FTO glasses were preliminarily cleaned by ultrasound in a solution of acetone and then ethanol for 10 min, after which they were dried in a thermostat at a temperature of 60 °C. The pre-swollen cryogel and its complexes with PEDOT were frozen and cut into thin layers (no more than 1 mm); the layer was spread on FTO glass and dried at 25 °C for 24 h.

### 3.10. Statistics

Experiments were performed with n = 3–4. All data measurements are represented as means ± standard deviations.

## 4. Conclusions

Polymerization of 3-sulfopropyl methacrylate or sulfobetaine methacrylate with N,N′-methylene-bis-acrylamide and functional comonomers (2-hydroxyethyl methacrylate, vinyl acetate) under cryotropic gelation conditions makes it possible to obtain porous polyelectrolyte gels. Introduction of vinyl acetate during the polymerization of sulfobetaine methacrylate leads to the formation of a cryogel, the degree of swelling of which is minimal in the presence of formic acid. The P(SPM-VA) gel (Young’s modulus, 0.1 MPa) showed the best strength properties, while the P(SBMA-VA) gel turned out to be the most elastic. Use of cryogels as a template during EDOT polymerization leads to the formation of cryogel@PEDOT complexes. It has been shown that when cryogels based on a copolymer of sulfobetaine methacrylate are used as a polyelectrolyte template, EDOT polymerization in the presence of formic acid leads to the formation of a PEDOT with polaron structure (a so-called quinoid structure). It has been established that electrochemical properties of cryogel@PEDOT complexes with a minimum degree of swelling are constant and do not depend on diffusion processes.

## Figures and Tables

**Table 1:** Sample acronyms and description.

Acronym	Description: Material Composition and Form
P(SPM-VA)	Synthesized by cryotropic gelation P(SPM-VA), gel
P(SPM-HEMA)	Synthesized by cryotropic gelation P(SPM-HEMA), gel
P(SBMA-VA)	Synthesized by cryotropic gelation P(SBMA-VA), gel
P(SBMA-HEMA)	Synthesized by cryotropic gelation P(SBMA-HEMA), gel
P(SPM-VA)@PEDOT	PEDOT synthesized within P(SPM-VA), gel
P(SBMA-VA)@PEDOT	PEDOT synthesized within P(SBMA-VA), gel
P(SBMA-HEMA)@PEDOT	PEDOT synthesized within P(SBMA-HEMA), gel

**Table 2:** Characteristics of cryogels based on SPM and SBMA.

Sample	Swelling Ratio, g/g		Gel Fraction, %	S, m^2^/g	D _Mesopore_, nm	Young’s Modulus, Pa
	H_2_O	HCOOH (96%)				
P(SPM-VA)	150 ± 6	95 ± 15	93	0.8	8–15	0.1
P(SPM-HEMA)	204 ± 40	61 ± 9	93	0.9	9–20	-
P(SBMA-HEMA)	20 ± 4	50 ± 6	81	1.3	7–10	6.3×10^−5^
P(SBMA-VA)	10 ± 2	43 ± 5	77	5	4–5	4.6×10^−6^

SEM images of cryogels obtained are shown in Figure 1.

## Data Availability

Not applicable.

## Supplementary Materials

The following supporting information can be downloaded at https://www.mdpi.com/article/10.3390/molecules27217576/s1: Figure S1: EDX-analysis of P(SPM-VA); Figure S2: EDX-analysis of P(SBMA-VA); Figure S3: EIS of P(SPM-VA); Figure S4: CVA of P(SBMA-HEMA); Figure S5: CVA of P(SBMA-VA); Figure S6: The simulation circuit of Rct in EIS analysis.
