# Supplementary material for: Influence of the Nature and Structure of Polyelectrolyte Cryogels on the Polymerization of (3,4-Ethylenedioxythiophene) and Spectroscopic Characterization of the Composites
Source: Molecules. 2022 Nov 4;27(21):7576. doi: 10.3390/molecules27217576 (PMC9659225; doi:10.3390/molecules27217576)
Supplement: Supplementary file 1 [file molecules-27-07576-s001.zip › molecules-2012302-supplementary.pdf]

molecules

Supplementary Materials

Article

Influence of the nature and structure of polyelectrolyte cryogels on the polymerization of (3,4-ethylenedioxythiophene) and electrochemical performance of the composites

Svetlana Laishevkina [1], Natalia Shevchenko [1]*, Olga Iakobson [1], Anatoly Dobrodumov [1], Vladimir Chelibanov [2] and Elena Tomšík [3]

[1] Institute of Macromolecular Compounds, Russian Academy of Sciences, Bolshoy pr., 31, Saint-Petersburg, 199004, Russia
[2] State University of Information Technologies, Mechanics and Optics, Kronverksky Pr. 49, bldg. A, St. Petersburg, 197101, Russia
[3] Institute of Macromolecular Chemistry AS CR, Heyrovsky Sq. 2, Prague, Czech Republic
* Correspondence: natali.shevchenko29@gmail.com (N.S.) tomsik@imc.cas.cz (E.T.).

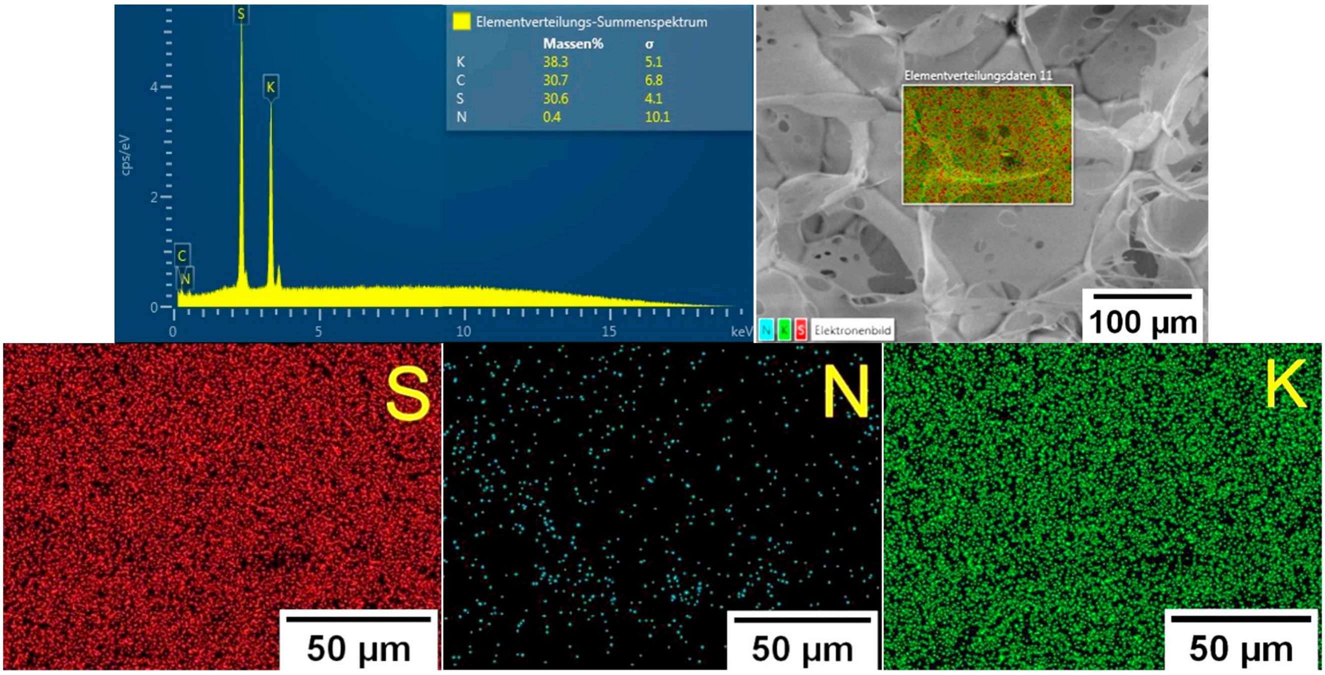

Figure S1. EDX-analysis of P(SPM-VA)

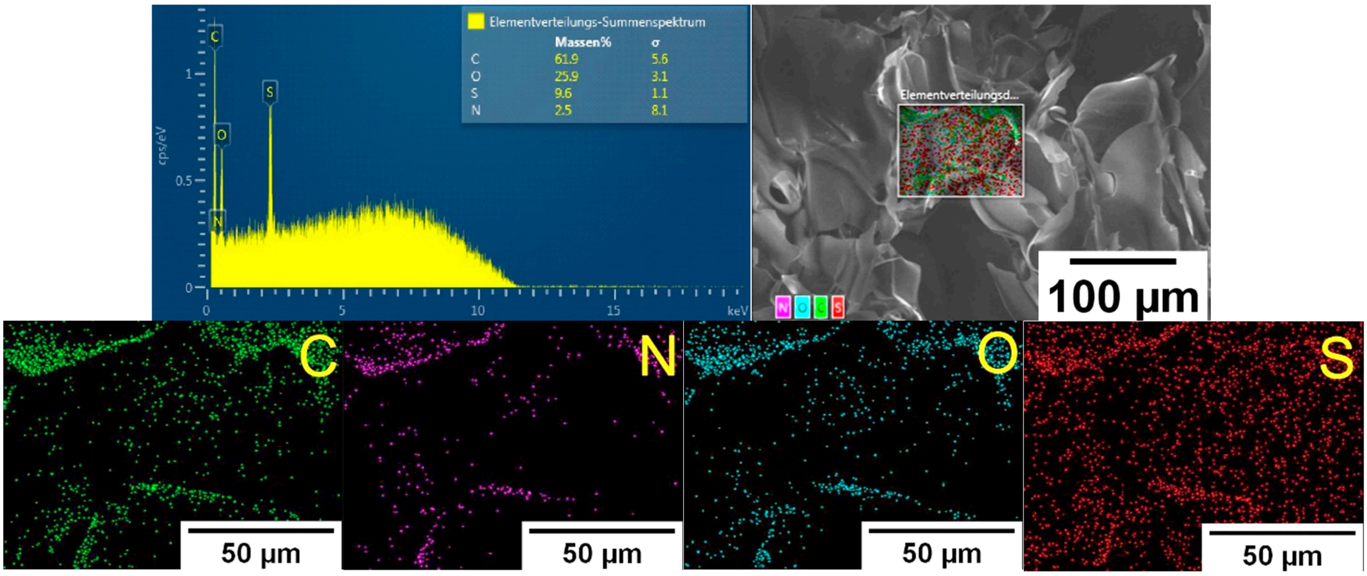

Figure S2. EDX-analysis of P(SBMA-VA)

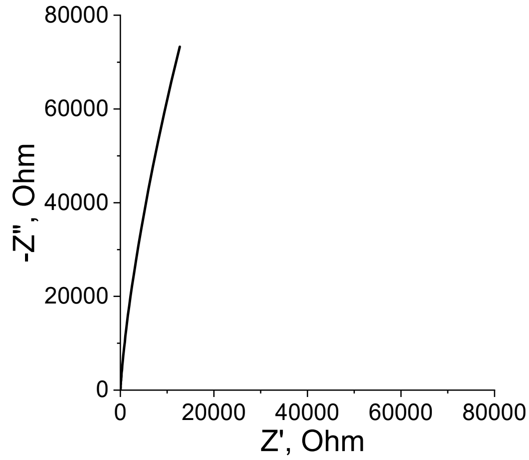

Figure S3. EIS of P(SBMA-VA)

Measurement of mechanical properties of cryogel by dynamic mechanical analyzer (DMA)

Before the compression test, the cryogels were immersed in distilled water for 15 min to achieve equilibrium swelling. Compression tests were performed using a tension compression test machine (DMA Q800 V21.3 Build 96) using samples of cylindrical shape (~10 mm diameter and ~10 mm height). Samples were compressed with two parallel plates at the maximum loading of 0.01 N with a compression rate of 1 mm/min. The stress, strain (%), and toughness values were calculated by the Trapezium X Materials Testing Software (Shimadzu, Japan). The compressive modulus was calculated from the linear region of the stress–strain curve (0–25% strain).

Cyclic voltammetry (CV)

CV measurements were done in three-electrode cell configuration in the potential window from -0.1 V to 0.8 V vs Ag/AgCl (3M KCl) reference electrode in 0.1 M H_2SO_4 with the scan rate from 10 mV/s to 150 mV/s.

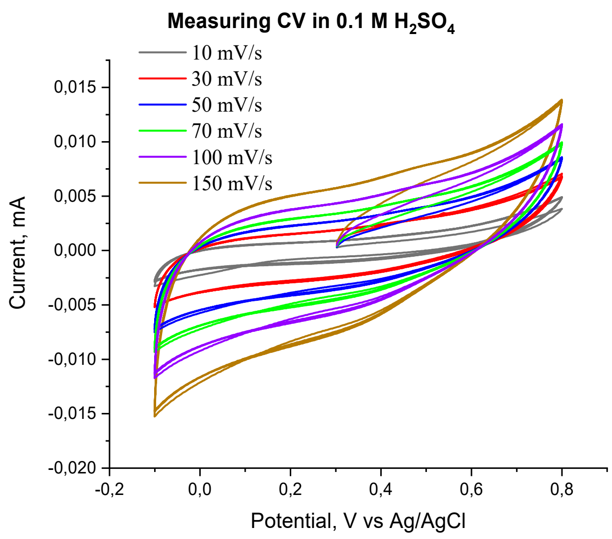

Figure S4. CVA of P(SBMA-HEMA)

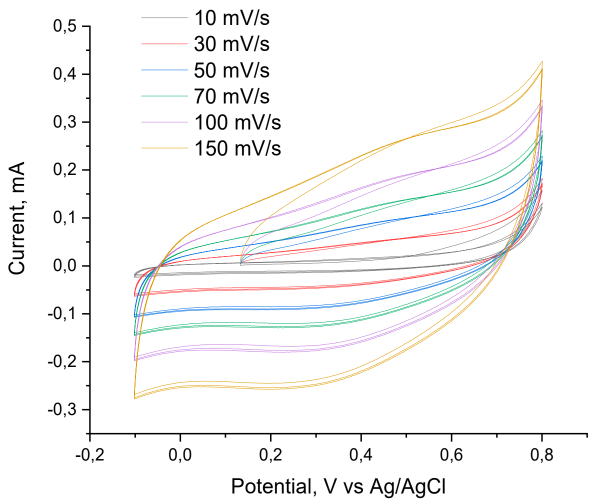

Figure S5. CVA of P(SBMA-VA)

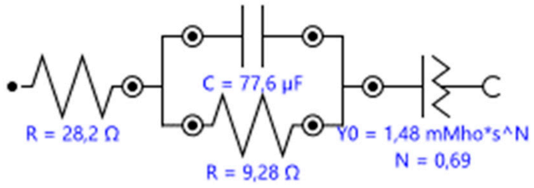

Figure S6. The simulation circuit of Rct in EIS analysis.

Element	Parameter	Value	Estimated Error (%)
R1	R	28,207	0,903
C1	C	7,7573E-05	9,977
R2	R	9,2752	6,264
Q1	Y0	0,0014812	1,031
	N	0,69028	0,676
	χ^2	0,19122	
